# Characterization of an Immunodominant Epitope in the Endodomain of the Coronavirus Membrane Protein

**DOI:** 10.3390/v8120327

**Published:** 2016-12-10

**Authors:** Hui Dong, Xin Zhang, Hongyan Shi, Jianfei Chen, Da Shi, Yunnuan Zhu, Li Feng

**Affiliations:** 1 State Key Laboratory of Veterinary Biotechnology, Division of Swine Infectious Diseases, Harbin Veterinary Research Institute, Chinese Academy of Agricultural Sciences, Harbin 150001, China; donghui_12215084@126.com (H.D.); zhangxin2410@163.com (X.Z.); shy2005y@163.com (H.S.); chenjianfei@126.com (J.C.); dashi198566@163.com (D.S.); zyn18345038162@163.com (Y.Z.); 2 Molecular Biology (Gembloux Agro-Bio Tech), University of Liège (ULg), Liège 4000, Belgium

**Keywords:** immunodominant epitope, coronavirus, membrane protein, endodomain

## Abstract

The coronavirus membrane (M) protein acts as a dominant immunogen and is a major player in virus assembly. In this study, we prepared two monoclonal antibodies (mAbs; 1C3 and 4C7) directed against the transmissible gastroenteritis virus (TGEV) M protein. The 1C3 and 4C7 mAbs both reacted with the native TGEV M protein in western blotting and immunofluorescence (IFA) assays. Two linear epitopes, 243YSTEART249 (1C3) and 243YSTEARTDNLSEQEKLLHMV262 (4C7), were identified in the endodomain of the TGEV M protein. The 1C3 mAb can be used for the detection of the TGEV M protein in different assays. An IFA method for the detection of TGEV M protein was optimized using mAb 1C3. Furthermore, the ability of the epitope identified in this study to stimulate antibody production was also evaluated. An immunodominant epitope in the TGEV membrane protein endodomain was identified. The results of this study have implications for further research on TGEV replication.

## 1. Introduction

Coronaviruses (CoVs) are clustered in the *Coronavirinae* subfamily and are divided into four genera (alpha-, beta-, gamma-, and deltacoronavirus) [1,2]. CoVs are enveloped, single-stranded, positive-sense RNA viruses [3,4,5]. The CoV genomes range from 26.2 kb to 31.7 kb in size. Four structural proteins are encoded by the CoV genomes: spike (S), membrane (M), envelope (E), and nucleocapsid (N).

Transmissible gastroenteritis virus (TGEV) is an excellent model of CoV biology [6,7,8,9,10,11,12]. The M protein is the viral assembly scaffold and the most abundant protein in the viral envelope [13]. The avian infectious bronchitis virus (IBV) M protein contains Golgi-targeting information in its first transmembrane domain [14], whereas the transmembrane domains and the cytoplasmic tail domain of the mouse hepatitis virus (MHV) M protein play important roles in Golgi targeting [15,16]. The M protein interacts with the E, S, and N proteins and plays an essential role in virus assembly [17,18,19]. M is a necessary component of virus-like particles (VLP) during viral assembly [18,20,21,22]. The M proteins interact other M proteins to form homo-oligomers [23]. In MHV, the M protein interacts with S, and deletion of the cytoplasmic tail of the M protein abolishes the effective interaction between the two proteins [24,25]. Interactions between the M and S proteins have also been identified in IBV [26], bovine coronavirus [27], and severe acute respiratory syndrome (SARS)-CoV [17,21].

The CoV M protein plays an important role in virion morphogenesis [28]. The M protein is composed of the following three regions: a small extracellular domain (ectodomain), a transmembrane domain (Tm), and a large carboxyl terminal domain (endodomain) [29]. The signal peptide of the M protein is located at amino acids (aa) 1–16 [30]. A single tyrosine in the M protein cytoplasmic tail is important for efficient interaction with the S protein of SARS-CoV [13]. The M protein of SARS CoV is localized in the endoplasmic reticulum (ER), Golgi, and ER Golgi intermediate compartment (ERGIC) [31,32]. The cytoplasmic tail of the CoV M protein is essential for its retention in the Golgi [16]. Current diagnostic tools for TGEV detection usually rely on PCR, and a specific method of indirect immunofluorescence assay (IFA) for TGEV detection is needed. TGEV M protein epitopes have been reported previously [28,33], but few functional studies have examined the cytoplasmic terminal domain (endodomain) of the CoV M protein. Monoclonal antibodies (mAbs) to the M protein are needed to dissect the function of the CoV M protein cytoplasmic tail.

In this study, the 1C3 and 4C7 mAbs against the TGEV M protein cytoplasmic tail are described. Two linear epitopes, 243YSTEART249 (1C3) and 243YSTEARTDNLSEQEKLLHMV262 (4C7), were identified in the M protein endodomain. An immunodominant epitope (aa 243–262) in the TGEV membrane protein endodomain was identified. The results of this study have implications for further research on TGEV replication.

## 2. Materials and Methods

### 2.1. Cells, Antibodies, and Virus

Porcine kidney 15 (PK-15) cells and Vero E6 cells were grown in DMEM medium supplemented with 10% fetal calf serum (5% CO_2_ and 37 °C). TGEV infectious strain H (Accession No. FJ755618) was propagated on PK-15 cells. Porcine epidemic diarrhea virus (PEDV) strain CV777 (Accession No. AF353511), the mAb against N protein of PEDV, and the mAb against N protein of TGEV were maintained in our lab. PEDV strain CV777 was propagated on Vero E6 cells.

### 2.2. Recombinant Plasmid Construction and Recombinant Protein Expression

The pCold-TGEV-M plasmid was constructed using the F-GST-M and R-GST-M primers (Table 1). Seven partial TGEV M genes corresponding to M protein amino acids (aa) 17–76 (nt 49–228), aa 67–126 (nt 199–378), aa 117–176 (nt 349–528), aa 167–226 (nt 499–678), aa 217–262 (nt 649–789), aa 217–246 (nt 649–738), and aa 234–262 (nt 700–789) were amplified with the primers shown in Table 1, which contained the *Bam* HI and *Xho* I restriction enzyme sites. The PCR products were cloned into the prokaryotic expression plasmid pGEX-6p-1. The recombinant plasmids were named pGEX GST-M1 (aa 17–76), pGEX GST-M2 (aa 67–126), pGEX GST-M3 (aa 117–176), pGEX GST-M4 (aa 167–226), pGEX GST-M5 (aa 217–262), pGEX GST-M6 (aa 217–246), and pGEX GST-M7 (aa 234–262).

### 2.3. Preparation of mAbs Targeting the M Protein

Proteins were expressed in *E. coli* BL21 (DE3) using previously described methods [34]. The GST-M fusion protein was purified using Glutathione Sepharose 4B (GE Healthcare, Amersham, UK) according to the manufacturer’s protocol. The mAbs against the M protein were prepared as previously described [35]. The SBA Clonotyping System-Horseradish Peroxidase (HRP) kit (Southern Biotechnology Associates, Inc., Birmingham, AL, USA) was used to determine the IgG subtype of the mAbs.

### 2.4. Immunofluorescence Assay (IFA)

PK-15 cells were infected with the TGEV H strain at a multiplicity of infection (MOI) of 0.1 and cultured for 36 h. The cells were fixed for 30 min with paraformaldehyde (4%) at 4 °C. The fixed cells were blocked with 5% skimmed milk and then incubated with the 1C3 or 4C7 mAb for 60 min at 37 °C. The cells were incubated with the anti-mouse IgG (whole molecule) Atto 488 antibody (1:1000, Sigma, St. Louis, MO, USA) after washing three times with 0.05% Tween 20 in PBS (PBST). Nuclear staining was performed with 4′,6-diamidino-2-phenylindole (DAPI, Sigma) [36]. The cells were washed three times with PBST and examined using a Leica TCS SP5 laser confocal microscope.

### 2.5. Immunoperoxidase Monolayer Assay (IPMA)

PK-15 cells were infected with the TGEV H strain and then fixed and blocked as described above. Then, the cells were incubated with the 1C3 or 4C7 mAb for 60 min at 37 °C. The cells were washed three times with PBST and incubated with HRP-labeled goat anti-mouse IgG (1:500, Sigma, USA) at 37 °C for 60 min. The cells were visualized with the 3-amino-9-ethylcarbazole (AEC) substrate and examined by microscopy.

### 2.6. Immunoprecipitation of the TGEV M Protein

Immunoprecipitation was performed as previously described [34]. The lysate from TGEV-infected or mock-infected PK-15 cells was incubated with 1 µg of the 1C3 or 4C7 mAbs at 4 °C. Protein A/G PLUS-Agarose was used according to the manufacturer’s instructions, and 60 μg of cell lysates was loaded in the gels. The immunoprecipitated proteins were analyzed by western blotting using the 1C3 or 4C7 mAbs as described previously [34].

### 2.7. Polypeptide Design and Coupling

Ten peptides spanning aa 217–262 of the TGEV M protein were synthesized by GL Biotech (Shanghai, China) (Table 2). Additionally, 4 mg of the RS-15 (RGDYSTEARTGGGGS), YT-16 (YSTEARTGGYSTEART), and YV-20 (YSTEARTDNLSEQEKLLHMV) peptides coupled with KLH (RS-15-KLH, YT-16-KLH, and YV-20-KLH) or BSA (RS-15-BSA, YT-16-BSA, and YV-20-BSA) were synthesized by GL Biotech.

### 2.8. Animal Immunization with RS-15-KLH, YT-16-KLH, and YV-20-KLH

Four BALB/c mice were immunized subcutaneously (s.c.) with RS-15-KLH, YT-16-KLH, or YV-20-KLH (100 μg per mouse) emulsified in complete Freund’s adjuvant (Sigma). The mice were immunized four times at two-week intervals. The sera were evaluated using ELISA plates coated with RS-15-BSA, YT-16-BSA, or YV-20-BSA (2 μg/well).

### 2.9. Peptide ELISA

ELISA plates were coated with the synthesized RS-15-BSA, YT-16-BSA, or YV-20-BSA peptide (2 μg/well) overnight at 4 °C and then blocked with 5% skimmed milk for 2 h at 37 °C. The plates were incubated with sera from mice immunized with RS-15-KLH, YT-16-KLH, or YV-20-KLH for 1 h at 37 °C. HRP-labeled goat anti-mouse IgG (1:2000, Sigma) was added and incubated for 1 h at 37 °C. The reaction was stopped with 2M H_2_SO_4_.

### 2.10. Immunohistochemistry (IHC)

The IHC assay was performed as previously described [37]. Slides were incubated with the 1C3 or 4C7 mAb (1:100) overnight at 4 °C, followed by incubation with HRP-labeled goat anti-mouse IgG (1:2000, Sigma) for 1 h at 37 °C. The reactions were detected with 3,3′-diaminobenzidine tetrahydrochloride (DAB) substrate.

### 2.11. 3D Epitope Modelling

The spatial distribution of the identified epitopes in the TGEV M protein were analyzed using PyMOL software with the SWISS-MODEL server [38].

### 2.12. Animal Ethics

This study was approved by Harbin Veterinary Research Institute and was performed in accordance with animal ethics guidelines and approved protocols. The animal Ethics Committee approval number is Heilongjiang-SYXK-2006-032.

## 3. Results

### 3.1. Expression and Purification of the GST-M Protein

**Figure 1:**
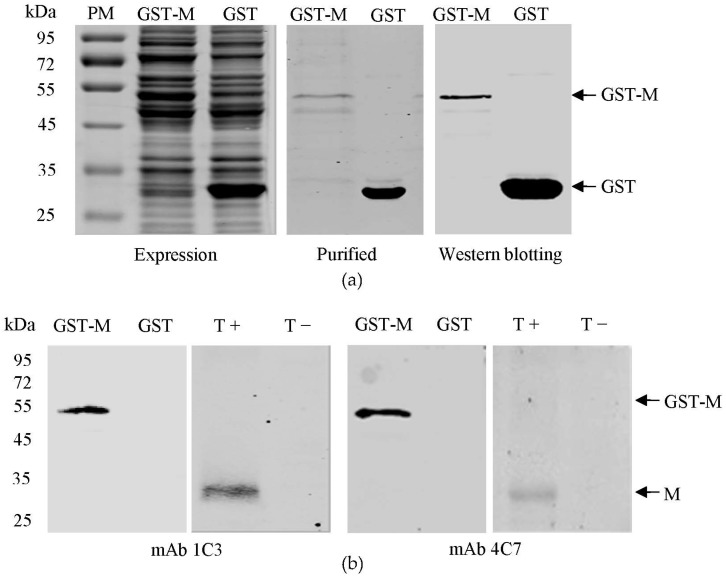
Preparation of monoclonal antibodies (mAbs) against the M protein of TGEV. (**a**) Expression and purification of GST-M protein. The proteins were detected after western blotting with a GST mAb; (**b**) Reactivity of the 1C3 and 4C7 mAbs with the GST-M protein and the TGEV M protein. PM represents protein marker. T+ represents the cell lysates of TGEV-infected porcine kidney 15 (PK-15) cells. T− represents the cell lysates of mock infected PK-15 cells.

For prokaryotic expression of the M protein, the signal peptide (aa 1–16) was removed, and the M gene was cloned into the prokaryotic expression vector pCold GST DNA. The recombinant proteins were expressed by induction with 1 mM IPTG in pCold-TGEV-M-transformed cells. The size of the recombinant GST-M protein was approximately 54 kDa. The purified GST-M protein reacted with the anti-GST mAb in the western blotting experiment (Figure 1a).

### 3.2. Preparation of mAbs against the TGEV M Protein

Two mAbs against the TGEV M protein (1C3 and 4C7) were prepared using the purified GST-M protein. The 1C3 and 3D7 mAbs belonged to the IgG2b isotype. As shown in Figure 1b, the 1C3 and 4C7 mAbs specifically reacted with both the GST-M protein and the native M protein in TGEV-infected PK-15 cells but not with GST and mock-infected PK-15 cells.

### 3.3. Determination of the 1C3 and 4C7 mAb Epitopes

**Figure 2:**
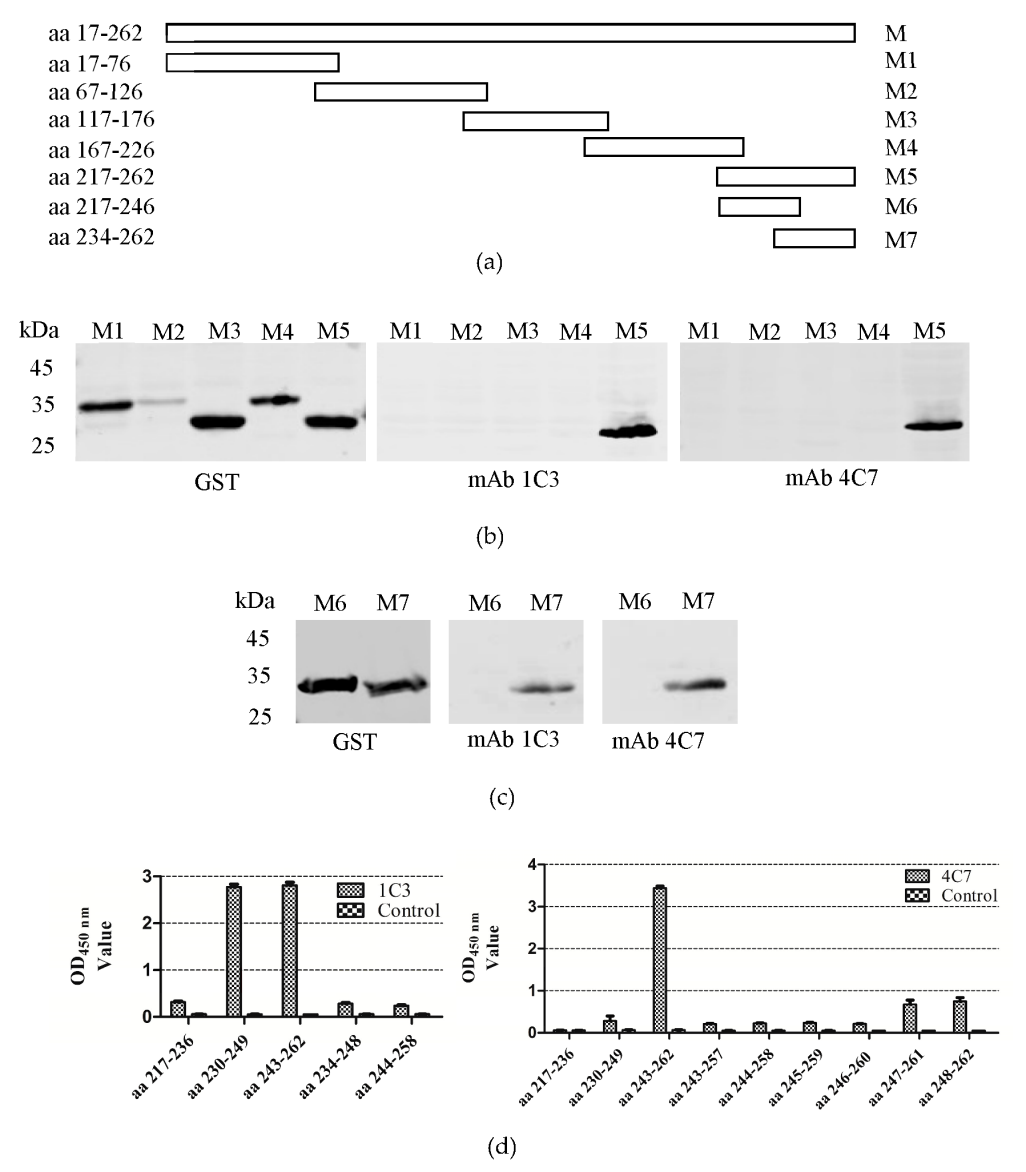
Identification of the epitopes of the 1C3 and 4C7 mAbs. (**a**) Scheme of the M protein and M fragments; (**b**) Western blotting analysis of the GST-M1, GST-M2, GST-M3, GST-M4, and GST-M5 proteins using the 1C3 and 4C7 mAbs; (**c**) Western blotting analysis of the GST-M6 and GST-M7 proteins using the 1C3 and 4C7 mAbs; (**d**) Five peptides were reacted with the mAb 1C3 and nine peptides with 4C7 by peptide ELISA. aa represents amino acids. PM represents protein marker.

To identify the 1C3 and 4C7 mAb epitopes, five truncated M proteins (GST-M1, GST-M2, GST-M3, GST-M4 and GST-M5) were expressed (Figure 2a). Figure 2b shows that 1C3 and 4C7 were reactive with GST-M5. Subsequently, two truncated M proteins that covered aa 217–262 were expressed. The western blotting results demonstrated that both 1C3 and 4C7 reacted against GST-M7 aa 234–262 (Figure 2c).

To further define the 1C3 and 4C7 mAb epitopes, ten overlapping polypeptides were synthesized (Table 2). The epitope ELISA results showed that 243YSTEART249 were the core amino acids of the 1C3 epitope, whereas 243YSTEARTDNLSEQEKLLHMV262 were the core amino acids of the 4C7 epitope (Figure 2d).

### 3.4. 3D Epitope Mapping

**Figure 3:**
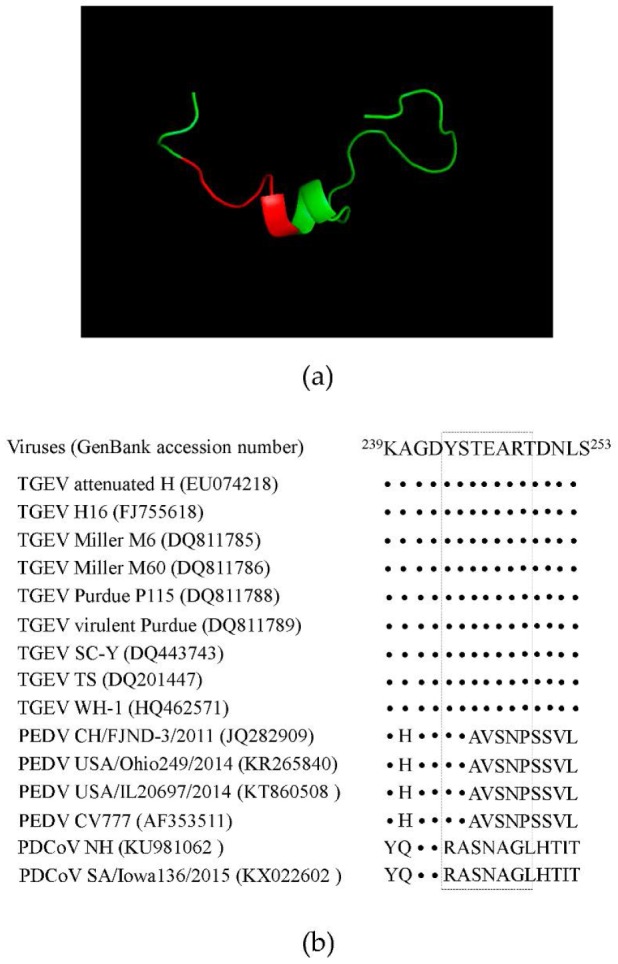
Location of the identified epitope in the predicted structure of the TGEV M protein. (**a**) The location of the epitope (shown in red) for mAb 1C3 (YSTEART) in the TGEV M protein is highlighted; (**b**) Conservation of the M epitopes (YSTEART) in TGEV, porcine epidemic diarrhea virus (PEDV) and porcine deltacoronavirus (PDCoV). Dots indicate identical residues.

The TGEV M protein sequence was compared against the SWISS-MODEL template library. The solution structure of ADP-ribosyl cyclase (SMTL id 1r15.1) [39] was selected for model building. The identified epitope recognized by mAb 1C3 (YSTEART) formed an alpha spiral structure (Figure 3a). Furthermore, the conservation of the M epitopes (YSTEART) in TGEV, PEDV, and porcine deltacoronavirus (PDCoV) was compared. As shown in the sequence alignment in Figure 3b, the epitope (YSTEART) is well conserved among TGEV, but differs greatly from the sequence in PEDV and PDCoV.

### 3.5. Reactivity of 1C3 and 4C7 with the TGEV M Protein in IFA and IPMA

**Figure 4:**
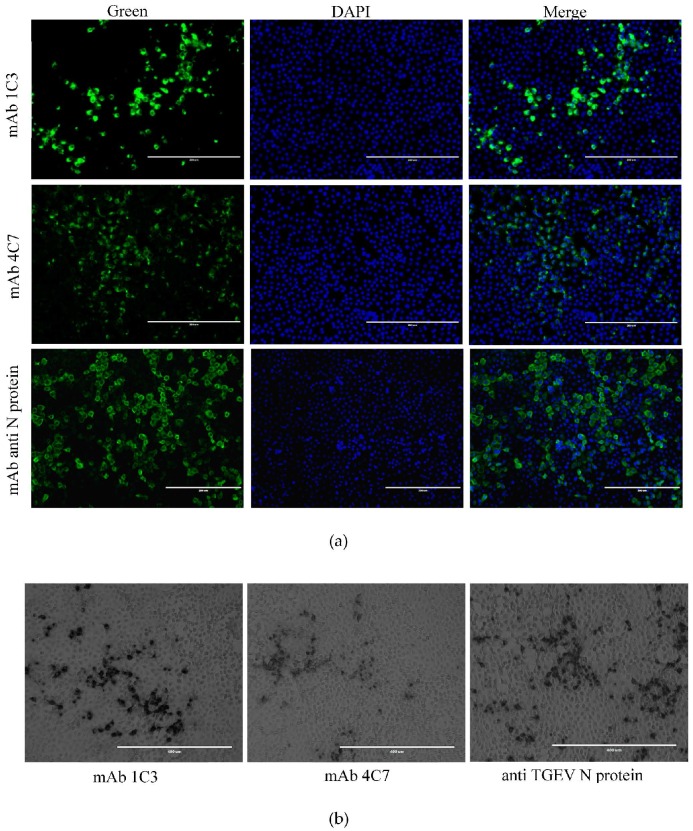
Application of the generated mAbs 1C3 and 4C7 in immunofluorescence assay (IFA) and immunoperoxidase monolayer assay (IPMA). (**a**) IFA analysis of the M protein in TGEV-infected PK-15 cells using 1C3 and 4C7 mAbs; (**b**) IPMA assay of the M protein in TGEV-infected PK-15 cells using 1C3 and 4C7 mAbs. The mAb against the N protein of TGEV was used as a positive control.

IFA and IPMA were used to verify the reactivity of mAbs 1C3 and 4C7 with the M protein in TGEV-infected PK-15 cells. The 1C3 and 4C7 mAbs showed reactivity with the M protein in TGEV-infected PK-15 cells in the IFA (Figure 4a) and IPMA (Figure 4b). The TGEV M protein was distributed in the cytoplasm of the PK-15 cells. The reaction ability of 1C3 was superior to 4C7 in the IFA and IPMA.

### 3.6. Immunoprecipitation of 1C3 and 4C7 with the TGEV M Protein

**Figure 5:**
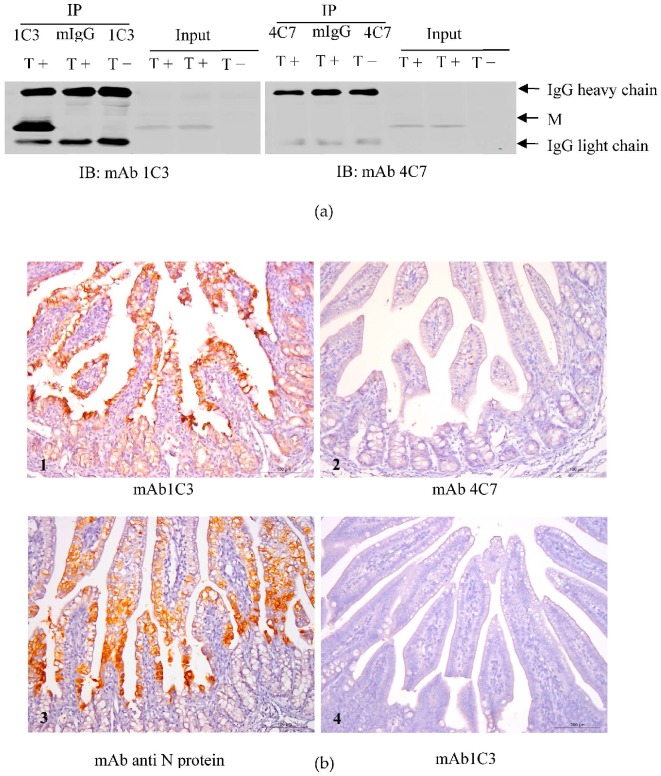
Application of the generated mAbs 1C3 and 4C7 in IP and immunohistochemistry (IHC). (**a**) Immunoprecipitation analysis of the M protein in TGEV-infected PK-15 cells using 1C3 and 4C7 mAbs. T+ represents the cell lysates of TGEV-infected PK-15 cells. T− represents the cell lysates of mock-infected PK-15 cells. The mIgG represents mouse control IgG; (**b**) IHC analysis of the M protein in the small intestines of TGEV-inoculated animals using 1C3 (1) and 4C7 (2) mAbs and an N-protein mAb (3) as a positive control. Staining of the small intestines of mock-inoculated animals with 1C3 mAb is shown as a negative control (4).

To elucidate whether the TGEV M protein could be precipitated with the 1C3 or 4C7 mAb, an immunoprecipitation assay was performed in the TGEV-infected PK-15 cells. As shown in Figure 5a, the TGEV M protein was precipitated from the TGEV-infected PK-15 cells by mAb 1C3 but not 4C7.

### 3.7. mAb 1C3 Reacted with the M Protein in the Small Intestine

The IHC assay was utilized to elucidate whether mAb 1C3 could recognize the M protein in the small intestines of animals inoculated with TGEV. As shown in Figure 5b, the TGEV M protein was recognized by mAb 1C3 but not 4C7 in TGEV-inoculated animal small intestines.

### 3.8. Optimizing of the IFA Method for the Detection of the M Protein

**Figure 6:**
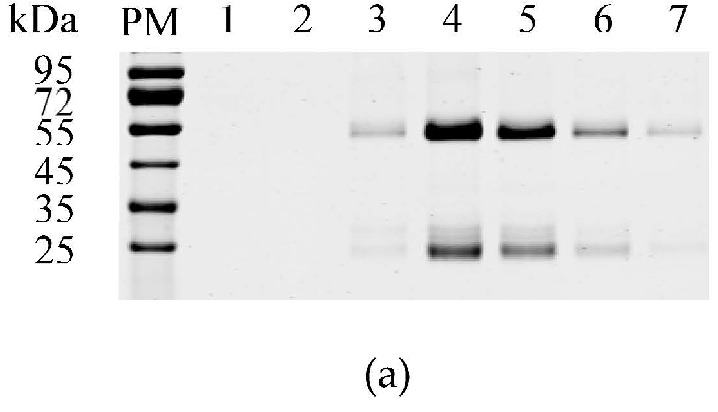

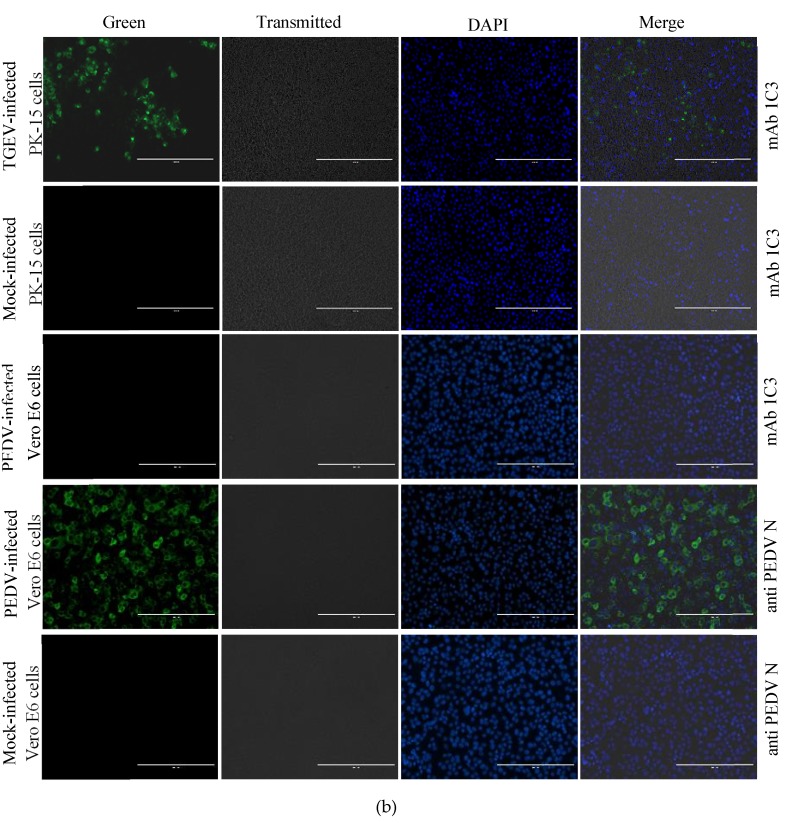
Optimization of the IFA method using mAb 1C3 for the M protein. (**a**) Purification of mAb 1C3 IgG. Lanes 1–7: purified IgG; (**b**) Optimization of the IFA method for M protein detection using the purified mAb 1C3 IgG in PK-15 cells. PM represents protein marker.

The IgG of mAb 1C3 was purified using HiTrapTM protein G HP (Figure 6a). The IFA method was optimized for the detection of the TGEV M protein. At 36 h, TGEV-infected PK-15 cells (10^3^ TCID_50_) were fixed with paraformaldehyde (4%) for 30 min at 4 °C. Then, the cells were blocked with 5% skimmed milk at 37 °C for 1 h. The optimum concentration of the primary antibody (purified 1C3 IgG) was 1 ng/µL, and the dilution of the secondary antibody was 1:500. The IFA detected green fluorescence in the TGEV-infected PK-15 cells (Figure 6b). To further validate whether 1C3 react with PEDV, IFA was used. As shown in Figure 6b, 1C3 did not react with the PEDV.

### 3.9. Antibody Responses to the Identified Epitopes

**Figure 7:**
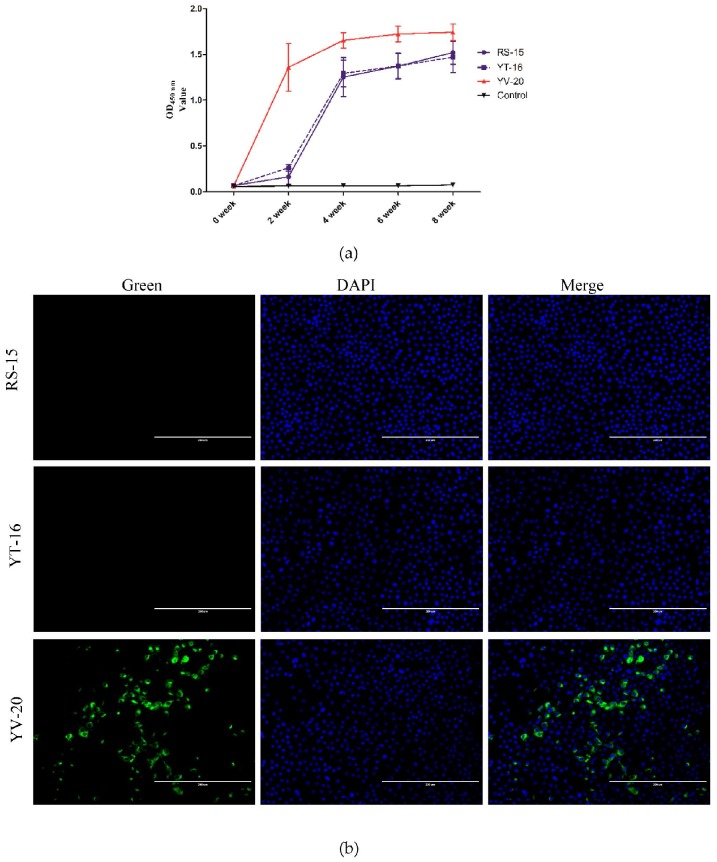
Antibody responses to the identified epitopes. (**a**) Humoral responses elicited by the aa 243-249 and aa 243–262 epitopes; (**b**) Reaction of antibodies elicited by epitopes with the TGEV virus in PK-15 cells.

To examine the ability of the two epitopes identified in this study to induce antibody responses, arginine-glycine-aspartate [40] was added on the N-terminal side of peptide aa 243–249 (RS-15), and an overlay of peptide aa 243–249 (YT-16) and peptide aa 243–262 (YV-20) were used to immunize mice. The epitopes were coupled with KLH and named RS-15-KLH, YT-16-KLH, and YV-20-KLH, respectively. BALB/c mice were immunized once every two weeks using RS-15-KLH, YT-16-KLH, or YV-20-KLH. Sera were collected at 0, 2, 4, 6 and 8 weeks. The antibodies elicited by RS-15-KLH, YT-16-KLH, and YV-20-KLH were detected using an indirect peptide ELISA with RS-15-BSA, YT-16-BSA, and YV-20-BSA as the antigen, respectively. At 4 weeks, the sera collected from the three immunized groups showed a detectable antibody response. In contrast, the control group inoculated with PBS did not show any significant immunity (Figure 7a). The antibody level increased with the number of immunizations. At 8 weeks, the antibody levels of all three immunized groups reached the highest values. Next, we examined whether the antibody was able to react with the native M protein in TGEV-infected PK-15 cells. Figure 7b shows that only the antibody elicited by the YV-20-KLH epitope reacted with the M protein, whereas no reaction was detected for RS-15-KLH or YT-16-KLH.

## 4. Discussion

The mapping of CoV viral protein epitopes can promote our understanding of the structure and function of the antigen. The CoV M protein is a major player in virus assembly [41], although its biology has not been fully elucidated. Monoclonal antibodies against the M protein are necessary to elucidate its various functions and mechanisms in viral replication. Some immunodominant epitopes have been identified on the M proteins (aa 193–200) of the porcine epidemic diarrhoea virus (PEDV) [42], IBV (aa 199–206) [43] and SARS-CoV (aa 1–31 and aa 132–161) [44]. Additionally, a few studies have reported monoclonal antibodies against the TGEV M protein [45,46,47]. However, no study has reported the TGEV M protein epitopes. In this study, two mAbs against the TGEV M protein (1C3 and 4C7) were prepared. Two epitopes recognized by mAbs 1C3 and 4C7 corresponding to 243YSTEART249 and 243YSTEARTDNLSEQEKLLHMV262 in the TGEV M protein were identified for the first time through a combination of experiments with truncated M proteins (M1–M7) and the peptide scanning technique. These results may indicate that the major immunodominant domain is located in the M protein endodomain.

Based on the peptide ELISA results, mAb 1C3 did not react with aa 244–258 and aa 234–248, indicating that Y243 and T249 were key residues for the activity of 243YSTEART249. The mAb 4C7 did not react with aa 243–257, aa 244–258, aa 245–259 or aa 246–260, which indicated that R248 and M261 were key residues for the activity of 243YSTEARTDNLSEQEKLLHMV262. Furthermore, by comparing aa 247–261 and 248–262 with aa 243–262, we found that the reactive activity of aa 243–262 was significantly higher than the reactive activity of the other amino acids (Figure 2d).

The CoVs M protein is a transmembrane protein with three domains: a small extracellular domain (ectodomain), a transmembrane domain (Tm), and a large carboxyl terminal domain (endodomain) [29,41,48]. M protein self-interactions occur among the transmembrane domains [49,50]. The ectodomain of the CoV M protein plays an important role in interactions with other viral proteins, such as the N protein of MHV [51,52,53], SARS-CoV [54,55,56], and TGEV [28] and the S protein of MHV [25] and SARS-CoV [13]. TGEV M aa 233–257 (AYYVKSKAAGDYSTEARTDNLSEQEK), which contains the epitopes recognized by the mAbs 1C3 and 4C7 (underlined), is involved in M-N binding to allow virion morphogenesis [28]. The fact might be a problem for mAbs (1C3 and 4C7) performance in a diagnostic test. In this study, the identified linear epitopes of mAbs 1C3 and 4C7 were located in the TGEV M protein endodomain. Thus, this information could be widely used in future research on the function of this domain in TGEV.

The IFA method we established has some advantages, including simple operation and easy evaluation of the results. A specific IFA method for the detection of the TGEV M protein is still needed. In this study, an IFA method for the detection of the M protein of TGEV was optimized. Optimization of this IFA method will be helpful for future studies of the function of the M protein in the process of TGEV replication. In general, coronavirus diagnostics is based on the N protein, because it is the most abundant protein, is produced early during infection, and is highly immunogenic. For detection of the TGEV virus, the established assay has no advantage over other N protein-based assays. Further research is needed to establish an IFA method for the detection of TGEV based on the N protein.

CoV structural protein can induce virus-specific antibodies [57]. The CoV S protein is a class I fusion protein involved in attachment of the CoV surface to the host aminopeptidase N [2,58]. The S protein is presented as a trimer and mediates receptor binding, membrane fusion, and virus entry [59,60,61]. The S protein is the major target for neutralizing antibodies [62,63]. The M protein can induce neutralizing antibodies, but these antibodies are weaker than those induced by the S protein. The TGEV M protein endodomain is also exposed on the virion surface [33] and some mAbs directed against the TGEV M endodomain are weakly neutralizing [64]. Further study is needed to evaluate the neutralizing activity of the prepared antibodies (1C3 and 4C7). An antibody against the M protein was induced and used to detect CoV [47,65]. In this study, mice were immunized with RS-15, YT-16, and YV-20. The antibody induced by aa 243–262 exhibited higher activity than the antibodies induced by RS-15 and YT-16 (Figure 7a). Furthermore, the antibody to YV-20 reacted with the TGEV M protein in TGEV-infected PK-15 cells in the IFA assay. However, the antibody to RS-15 and YT-16 did not react with the M protein in TGEV-infected PK-15 cells (Figure 7b). These results indicate that the YV-20 epitope has potential for the development of a TGEV vaccine.

## 5. Conclusions

Two specific mAbs against the TGEV M protein (1C3 and 4C7) were prepared in this study, and two linear B cell epitopes located in the M protein endodomain were successfully identified. The 1C3 mAb was used to immunoprecipitate the M protein from TGEV-infected PK-15 cell lysates. The 1C3 mAb is a useful tool for investigations of the antigenic properties of the M protein. These antibodies are relevant to furthering our understanding of the mechanism of the M protein in TGEV replication. Furthermore, an immunodominant epitope (aa 243–262) in the TGEV membrane protein endodomain was identified.

## Figures and Tables

**Table 1:** Primers used in this study.

Name	Sequence	Enzyme
F-GST-M	CCGCTCGAGGAACGCTATTGTGC	*Xho* I
R-GST-M	CGGAATTCTTATACCATATGTA	*Eco* RI
F-M (49–228)-6p	GTGGATCCGAACGCTATTGTGCTATGAA	*Bam* HI
R-M (49–228)-6p	GACTCGAGGAATTGAGGTCTTCCATATT	*Xho* I
F-M (199–378)-6p	GTGGATCC ACTGTGCTACAATATGGAAG	*Bam* HI
R-M (199–378)-6p	GACTCGAGAAATGTAACAATTGCACCTG	*Xho* I
F-M (349–528)-6p	GTGGATCCTTTAGTATTGCAGGTGCAAT	*Bam* HI
R-M (349–528)-6p	GACTCGAGACCAGTTGGCACACCTTCGA	*Xho* I
F-M (499–678)-6p	GTGGATCCGTGCTTCCTCTCGAAGGTGT	*Bam* HI
R-M (499–678)-6p	GACTCGAGTGCTTTCAACTTCTTGCCAA	*Xho* I
F-M (649–789)-6p	GTGGATCCTACACACTTGTTGGCAAGAA	*Bam* HI
R-M (649–789)-6p	GACTCGAGTTATACCATATGTAATAATT	*Xho* I
F-M (649–738)-6p	GTGGATCCTACACACTTGTTGGCAAGAA	*Bam* HI
R-M (649–738)-6p	GACTCGAGCTCTGTTGAGTAATCACCAG	*Xho* I
F-M (700–789)-6p	GTGGATCCTACTATGTAAAATCTAAAGC	*Bam* HI
R-M (700–789)-6p	GACTCGAGTTATACCATATGTAATAATT	*Xho* I

**Table 2:** Synthesized polypeptides based on the M protein of the transmissible gastroenteritis virus (TGEV).

Residues	Amino Acid Sequence	Residues	Amino Acid Sequence
217–236	YTLVGKKLKASSATGWAYYV	230–249	TGWAYYVKSKAGDYSTEART
243–262	YSTEARTDNLSEQEKLLHMV	234–248	YYVKSKAGDYSTEAR
243–257	YSTEARTDNLSEQEK	244–258	STEARTDNLSEQEKL
245–259	TEARTDNLSEQEKLL	246–260	EARTDNLSEQEKLLH
247–261	ARTDNLSEQEKLLHM	248–262	RTDNLSEQEKLLHMV
RS-15	RGDYSTEARTGGGGS	YT-16	YSTEARTGGYSTEART
YV-20	YSTEARTDNLSEQEKLLHMV		
